# Vaccine Therapy of Typhoid

**Published:** 1914-08

**Authors:** 


**Vaccine Therapy of Typhoid.** Albert A. Horner, Boston, M. & S. Jour., June 25, 1914. In a comparison of 40 cases treated with vaccines, ten million bacilli, increasing by ten million daily for a week, then 20 million on alternate days until the temperature had been normal for three days or until a total of 700 million was reached (average total dosage 516 million) with 95 cases treated without vaccines, no conspicuous differences were found. The febrile period was 24.39 days for the average of the vaccinated series, 23.08 for the unvaccinated; complications occurred in 55 to 50.5% respectively; mortality 10 and 11.5%; relapse 20 and 11.5%; haemorrhage 7.5 and 2.15%; pneumonia 10.25 and 4.15%; phlebitis 5 and 0%.

